# Genotyping of *Salmonella enterica* serovar Typhi strains isolated from 1959 to 2006 in China and analysis of genetic diversity by genomic microarray

**DOI:** 10.3325/cmj.2011.52.688

**Published:** 2011-12

**Authors:** Haifang Zhang, Xiaolei Zhang, Meiying Yan, Bo Pang, Biao Kan, Huaxi Xu, Xinxiang Huang

**Affiliations:** 1 Department of Biochemistry and Molecular Biology, School of Basic Medicine and Medical Technology, Jiangsu University, Zhenjiang, Jiangsu, China; 2 State Key Laboratory for Infectious Disease Prevention and Control, National Institute for Communicable Disease Control and Prevention, Chinese Center for Disease Control and Prevention, Beijing, China

## Abstract

**Aim:**

To determine the genotype of *Salmonella enterica* serovar Typhi (*S.* Typhi) strains in China and analyze their genetic diversity.

**Methods:**

We collected *S.* Typhi strains from 1959 to 2006 in five highly endemic Chinese provinces and chose 40 representative strains. Multilocus sequence typing was used to determine the genotypes or sequence types (ST) and microarray-based comparative genomic hybridization (M-CGH) to investigate the differences in gene content among these strains.

**Results:**

Forty representative *S.* Typhi strains belonged to 4 sequence types (ST1, ST2, ST890, and ST892). The predominant *S.* Typhi genotype (31/40) was ST2 and it had a diverse geographic distribution. We discovered two novel STs – ST890 and ST892. M-CGH showed that 69 genes in these two novel STs were divergent from *S.* Typhi Ty2, which belongs to ST1. In addition, 5 representative Typhi strains of ST2 isolated from Guizhou province showed differences in divergent genes.

**Conclusion:**

We determined two novel sequence types, ST890 and ST892, and found that ST2 was the most prevalent genotype of *S.* Typhi in China. Genetic diversity was present even within a highly clonal bacterial population.

*Salmonella enterica* serovar Typhi (*S.* Typhi) is a Gram-negative, human-restricted enteroinvasive pathogen that causes typhoid fever (1,2). The harm caused by *S.* Typhi has been greatly reduced by the application of antibiotics, but typhoid fever is still a common disease in tropical and subtropical regions, and many drug-resistant strains of *S.* Typhi have been discovered. In the recent years, there have been more than 16 million cases reported annually worldwide. Even in the United States and other developed countries, there are still outbreaks of typhoid fever caused by *S.* Typhi (3). This is in part due to the ability of *S.* Typhi to rapidly evolve through either horizontal gene transfer mechanisms or produce a cloud of related strains by using highly mutable genes (4). Thus, there is an urgent need for improved molecular diagnostics to discriminate among the large numbers of related strains. There are many methods for genotyping of *Salmonella,* and polymerase chain reaction (PCR)-based typing methods are very prevalent. A multiplex PCR-based reverse line blot hybridization system can enhance outbreak investigations and surveillance of *Salmonella* infections (5). Recently, real-time PCR-based single nucleotide polymorphism typing method has been used for global epidemiological analysis of *S.* Typhi (6).

Multilocus sequence typing (MLST), which is based on the analysis of DNA sequence polymorphisms in a group of housekeeping genes, is the most widely used method for bacterial strain genotyping (7). Each unique sequence of a housekeeping gene is assigned an allele number, and an allele profile of a strain is defined as the set of allele numbers for that strain. Each unique allele profile is assigned a sequence type (ST) number. Strains that have the same ST number are identical at all of the sequenced loci and are considered to be members of the same clone. MLST, unlike earlier molecular typing methods, such as pulsed-field gel electrophoresis (PFGE), has high discriminative power, allows easy standardization of data acquisition and analysis across laboratories, and has a high degree of portability of the resulting sequence data (8). MLST was used to investigate the genotype of *S.* Typhi as early as in 2002 (9). Up to now, 51 *S.* Typhi strains have been recorded in the *Salmonella enterica* MLST Database (10) and classified into 8 STs (ST1, ST2, ST3, ST8, ST890, ST892, ST911, and ST980), but most of them belong to ST1 (15/51) and ST2 (29/51).

One well accepted definition of procaryotic species, based on whole-genome DNA-DNA hybridization, is that it is an entity comprised of strains sharing a reassociation value of approximately 70% or greater (11). Genomic diversity and relatedness of closely related organisms has since recently been determined with microarrays, which have higher resolution than traditional DNA-DNA hybridization methods (12). Microarray-based comparative genomic hybridization (M-CGH) is in widespread use in relatedness determination of procaryotic species (13-15). CGH usually uses the whole genome open reading frame (ORF) array-based hybridization approach (16).

Typhoid fever is endemic in developing countries, such as China. However, there are few reports of genotyping of *S.* Typhi in China (17). These reports mostly used PFGE, which is currently the method of choice for genotyping of sporadic or epidemic *Salmonella* isolates. *S*. typhi strains isolated from Shenzhen in China showed 22 distinct PFGE patterns with variable genetic diversity (17). We speculated that genetic diversity of *S*. Typhi strains may be largely present throughout the last several decades in China. Therefore, we collected *S.* Typhi strains from 1959 to 2006 and chose 40 strains representative in terms of genetic diversity isolated from 5 highly endemic Chinese provinces. The aim of this study was to identify the genotype of 40 representative *S.* Typhi strains by MLST and evaluate their genetic diversity by M-CGH.

## Materials and methods

### Bacterial strains

We performed MLST genotyping and M-CGH analysis on 40 representative *S.* Typhi strains isolated from 5 highly endemic Chinese provinces – Xinjiang, Guangxi, Guizhou, Henan, and Zhejiang between 1959 and 2006, which are kept in the Chinese Center for Disease Control and Prevention, Beijing (web-extra material).

### Genomic DNA isolation

Bacteria were grown in Luria-Bertani broth at 37°C before genomic DNA isolation using the AxyPrep™ Bacterial Genomic DNA Miniprep Kit (Axygen Biosciences, Union City, CA, USA) following the manufacturer's instructions. All genomic DNA samples were treated with RNase A, subjected to electrophoresis in agarose gels, and stained with ethidium bromide before MLST. The concentration of DNA was determined with an ND-1000 spectrophotometer (NanoDrop Technologies, Wilmington, DE, USA).

### Multilocus sequence typing and analysis

MLST was performed as previously described (18). PCR amplification of the 7 housekeeping genes – *hisD*, *aroC*, *dnaN*, *hemD*, *purE*, *sucA,* and *thrA* was done with the primers shown in Table 1. The primers and settings are described on the MLST website (10). Amplicons were sequenced by Sangon Biotech Co., Ltd (Shanghai, China). MLST alleles and sequence types (ST) were determined for each strain by querying the *Salmonella enterica* MLST Database with the edited sequence data. Allele numbers and STs were assigned using the database and novel alleles and STs were submitted to the database.

**Table 1:** Primers used in multilocus sequence typing.of *Salmonella enterica* serovar Typhi (*S.* Typhi) strains isolated in China

Gene name	Primers	Length of polymerase chain reaction amplicon(bp)
*purE*	GCACCACAGGAGTTTTAAGA ACCTCATCGGTTTGTGCTTT	399
*hemD*	GCCCGCATGAGTATTCTGAT CAATAGCCGACAGCGTAGTA	432
*sucA*	CTGAGTATATGCACATCACC CACCTGGTTGTTGATAACGA	501
*aroC*	GCCTGCTGATTGAAAACACC AATGTGTTGCCCACTACTGA	501
*hisD*	GTCTGTCGGTCTGTATATTC GCCCATACTGATTAGAGATG	501
*dnaN*	CCGATCTTGAAATGGAGATG TTATTACTGCCGATCTGCAC	501
*thrA*	GCCATTACCTTGAATCTACC GCTCCAGATAGAACTCATCC	501

### Microarray-based comparative genomic hybridization and analysis

We used a genomic DNA microarray containing 4181 ORFs from the genome of S. Typhi Ty2. Details of this microarray, including primer selection, parameters for primer synthesis, selection of amplicons, as well as purification and printing of DNA onto slides, have been previously described (19).

### DNA isolation

*S.* Typhi Ty2 was used as the reference strain for M-CGH. Two S. Typhi strains of novel sequence types and 5 S. Typhi strains of ST2 isolated from Guizhou province in 1959, 1973, 1986, 1995, and 2006 were used as the test strains. Genomic DNA was isolated by MLST.

### Fluorescent labeling and hybridization

The genomic DNA of *S.* Typhi Ty2 was used as the reference DNA and genomic DNA from each of the *S.* Typhi isolates was used as the test DNA. Cy3- and Cy5-labeled probes were generated by priming the reference and test DNA with random hexamers and extension with Klenow fragment (20). The reference and test DNA were combined to hybridize to our microarray using protocols described elsewhere (21) and all hybridizations were done in duplicate. Pairwise comparisons were made for each strain using dye swaps to avoid labeling bias.

### Microarray data acquisition and analysis

The slides were scanned with a GenePix Personal 4100A microarray scanner (Molecular Devices, Sunnyvale, CA, USA) using two channels of appropriate lasers. GenePix Pro 6.0 (Molecular Devices) was used to process the scanning images and quantify the spot intensity. Spots with bad data due to slide abnormalities were discarded. Data normalization was done on the remaining spots by global normalization mode. Data were exported into Microsoft Excel for analysis as described before (21,22) with minor modifications. In brief, the ratio of reference DNA normalized intensity to test DNA normalized intensity was converted to log_2_ and the mean log_2_ ratio was calculated for the same gene from different slides. The threshold of the mean log_2_ ratio was set at 1.58 (a little greater than 3-fold) to represent divergent genes between the reference and test strains. On the basis of these results, if the intensity of spots for the test DNA was <1000, these spots were taken as the genes absent from the test strain compared to the reference strain.

## Results

### MLST genotypes

Partial sequences of 7 housekeeping genes revealed a low genetic variation; only 3 base substitutions were detected, yielding two multilocus genotypes or sequence types (ST890 and ST892) (Table 2). Thus, two novel alleles were identified: *hisD*353, which consists of a synonymous substitution compared to *hisD*1 allele present in most of the *S*. Typhi strains, and *aroC*305, which consists of a missense substitution compared to the predominant *aroC*1 allele. The novel sequence types, ST890 and ST892, were found only in Xinjiang Province and Guangxi Province, respectively. The predominant Typhi genotype was ST2 (31/40 Typhi) and it had a diverse geographic distribution. However, it is not known whether these *S*. Typhi strains of ST2 can be classified into subspecies.

**Table 2:** Allelic profiles and sequence types (ST) of 40 *Salmonella enterica* serovar Typhi strains isolated in China

Multilocus allelic profile*								Number of isolates	Provinces^†^	Years
ST	*aroC*	*dnaN*	*hemD*	*hisD*	*purE*	*sucA*	*thrA*			
1	1	1	1	1	1	1	5	6	XJ, GZ	1984-2005
2	1	1	2	1	1	1	5	31	GZ, GX, XJ, ZJ, HN	1959-2006
890^‡^	1	1	2	353^‡^	1	1	5	1	XJ	1999
892^‡^	305^‡^	1	2	1	1	1	5	2	GX	1998, 2004

*Allele and ST numbers are those assigned in the *Salmonella* multilocus sequence typing database (10).

†GZ – Guizhou; GX – Guangxi; XJ – Xinjiang; ZJ – Zhejiang; HN – Henan.

‡Novel alleles and STs obtained in this study.

### Genotyping by M-CGH

M-CGH experiments with two novel sequence types and *S.* Typhi Ty2 were done to assess strain-strain relationships on the basis of genome similarity. Many genes diverged into two novel sequence types compared to *S.* Typhi Ty2 (Table 3), and many of these genes were clustered in operons. In ST892, 69 genes were classified as divergent, 38 of which were absent and 31 had variable presence. In ST890, there were 28 divergent genes but only 9 were absent. It was surprising that genes *t2648, t2649, t2650, t2651,* and *t2652* were all absent from ST890 and ST892.

**Table 3:** The absent and divergent genes in two novel sequence types (ST) of *S.* Typhi compared to *S.* Typhi Ty2

	Novel sequence types	
	ST890	ST892
Absent genes	*t2648, t2649, t2650, t2651, t2652, t2653, t2654, t2655, t2656*	*t1134, t1135, t1137, t1141, t1151, t1691, t2648, t2649, t2650, t2651, t2652, t4259, t4260, t4261, t4263, t4264, t4265, t4266, t4268, t4269, t4270, t4271, t4273, t4274, t4275, t4281, t4282, t4283, t4284, t4286, t4289, nucD, nucE, t4317, t4318, t4319, t4320, t4321*
Variably present genes	*t3052, t3053, rpsO, ftsH, ftsJ, mreB, rhlB, rplJ, rplA, rplK, rplJ, rplA, atpB, atpH, atpA, atpG, atpC, t4354, t4355*	*pilL, pilM, pilO, pilP, pilT, pilU, t4326, t4326, t4328, t4329, t4330, t4331, t4332, t4333, t4334, vexE, vexD, vexC, vexA, tviE, tviD, tviB, t4354, t4355, t4356, t4357, t4358, t4359, t4361, t4363, t4366*

Five *S*. Typhi strains of ST2 isolated in 1959, 1973, 1986, 1995, and 2006 in Guizhou province were analyzed by M-CGH in order to identify genetic differences between them. There were many divergent genes in these 5 *S*. Typhi strains compared to *S.* Typhi Ty2, although they belong to the same MLST sequence type ST2 (Table 4). The divergent genes in each of these 5 strains were all different. Compared to *S.* Typhi Ty2, there were more divergent genes in the *S*. Typhi strains of ST2 isolated in 1959 than in other strains of ST2 isolated in 1973, 1986, 1995, and 2006; ie, there were differences in the genome content between the same MLST sequence type strains. Therefore, M-CGH could be used to classify the same MLST sequence types. In addition, an interesting finding was that genes *t2649, t2650, t2651, t2652, t2653,* and *t2654* were all absent from these 5 strains, very similar to what was found for ST890 and ST892.

**Table 4:** The absent and divergent genes in 5 *S*. Typhi strains of sequence type ST2 isolated in Guizhou province compared to *S.* Typhi Ty2

	Five *S*. Typhi strains of ST2 isolated in Guizhou Province				
Year	1959	1973	1986	1995	2006
Absent genes	*t2649, t2650, t2651, t2652, t2653, t2654, t2655, t2656, t4519, t4520, t4521, t4522, t4523, t4524, t4527, t4528*	*t2649, t2650, t2651, t2652, t2653, t2654,*	*t2649, t2650, t2651, t2652, t2653, t2654, t2655,*	*t0872, t2649, t2650, t2651, t2652, t2653, t2654, t2655*	*t2649, t2650, t2651, t2652, t2653, t2654, t2655, t4519, t4520, t4521, t4528*
Variably present genes	*t2647, t4226, t4227, t4228, t4229, t4230, t4231, t4232, t4233, topB, pilL, pilM, pilO, pilP, pilQ, pilR, pilS, pilU, pilV, t4328, t4329, t4330, t4331, t4332, t4333, t4334, vexE, vexD, vexB, vexA, t4354, t4355, t4356, t4357*	*t0869, t0872, t2948, t2949, t4953*	*t2647, t2648, t2656, t4521, t4528*	*t0869, t0871, t2647, t2648*	*t2647, t2648, t2656, t4518, t4522, t4523, t4525, t4527*

## Discussion

We used MLST to analyze 40 *S.* Typhi isolates from 5 Chinese provinces and found two novel sequence types, ST890 and ST892. In ST890, the housekeeping gene *hisD* consisted of a synonymous substitution that does not change the amino acid. In ST892, the point mutation of the housekeeping gene *aroC* was a missense mutation leading to the amino acid substitution Ala (hydrophobic) to Thr (hydrophilic).

The complete genome sequence of *S.* Typhi Ty2 has been determined (23) and its sequence type in the *Salmonella* MLST database is ST1 (10). Therefore, it was used as the reference strain, and a genomic DNA microarray based on the genome of Ty2 was used for M-CGH. There were many more absent and variably present genes in ST892 than in ST890; so, we speculate that the genetic variability of ST892 is greater than that of ST890. Moreover, the genetic variability of the same ST2 isolated in 1959, 1973, 1986, 1995, and 2006 in Guizhou province was different. Apparently, a large proportion of the absent and variably present genes of the tested strains consisted of hypothetical ORFs encoding unknown or unassigned functions, and this appears to be a common trait among procaryotes (15,20,24). It is very interesting that the genes absent from the strains ST890 and ST892 and 5 ST2 strains were located mostly in the gene cluster *t2648-t2656*. However, these genes were present in the ST1 strains isolated from Guizhou Province (M-CGH, data not shown). This gene cluster might be a specific genetic fragment in the ST1 strains of *S.* Typhi. It is reported that genes *t2648-t2656* are similar to some genes of bacteriophage P4 (25). The considerable gene acquisition/loss promoted the genetic diversification of *S.* Typhi (26,27). In this study, the genetic differences between 5 *S.* Typhi strains of ST2 isolated from Guizhou Province indicated that the genomic reservoir was unstable even within a highly clonal bacterial population. In particular, the common absent or divergent genes found by M-CGH are expected to be marker genes to demonstrate the genetic relationship between the strains. However, the functions of these absent or divergent genes found by M-CGH are not clear and need to be clarified in more experiments.

In summary, this study found that the genotypes of *S.* Typhi isolated from China between 1959 and 2006 belonged to 4 sequence types (ST1, ST2, ST890, and ST892) and found two novel sequence types, ST890 and ST892. It also demonstrated the extent of genetic variation of these strains throughout several decades in China.
